# Diabetic ketoacidosis knowledge in type 1 diabetes: a Brazilian perspective

**DOI:** 10.20945/2359-4292-2025-0036

**Published:** 2025-08-18

**Authors:** Renan Bruno Faria Pisani, Virgínia Valiate Gonzalez, Bruna Rambo Witte, Taylane Guimarães Gonçalves, Lohane Alves Santiago, Adalgiza Mafra Moreno, Rodrigo de Azeredo Siqueira

**Affiliations:** 1 Universidade Iguaçu, Nova Iguaçu, RJ, Brazil

**Keywords:** Diabetes mellitus, type 1, Diabetic ketoacidosis, Primary prevention, Patient education as topic

## Abstract

**Objective:**

To evaluate the factors associated with knowledge of diabetic ketoacidosis among individuals with type 1 diabetes mellitus and to analyze how sociodemographic, clinical, and healthcare access variables influence understanding of the condition’s prevention and management.

**Methods:**

This cross-sectional study was conducted with 465 individuals with type 1 diabetes mellitus via an online questionnaire. The instrument included sociodemographic and clinical variables and a 13-item test assessing participant’s knowledge of diabetic ketoacidosis. Data analysis employed the Kruskal-Wallis test and the Dwass-Steel-Critchlow-Fligner multiple comparisons to identify variability scores.

**Results:**

The median score was 6.0 (on a scale of zero to 13). The lowest accuracy rates were observed regarding ketonemia thresholds (18.5%) and specific warning signs of diabetic ketoacidosis (26.0%). Participants with prior knowledge of diabetic ketoacidosis scored higher (median 6) than those unfamiliar with the term (median 2). Individuals with glycated hemoglobin levels between 4 and 6% achieved higher scores (median = 6), as did those who received medical guidance (median = 6) compared to those who did not (median = 3). The use of an artificial pancreas was associated with the highest scores.

**Conclusion:**

Prior knowledge of diabetic ketoacidosis, access to insulin pumps, medical guidance, and better glycemic control were associated with a greater understanding of diabetic ketoacidosis. These findings reinforce the need for educational interventions and equitable access to healthcare technologies to improve disease management and reduce diabetic ketoacidosis-related complications.

## INTRODUCTION

Diabetic ketoacidosis (DKA) is an acute complication of type 1 diabetes mellitus (T1DM), associated with high rates of morbidity and mortality (^1^). Although largely preventable, DKA poses a significant challenge for healthcare systems worldwide. In the United States, the total cost of DKA-related hospitalizations increased by US$ 5.1 to 6.75 billion between 2014 and 2017, underscoring its growing economic impact (^2^). In least-developed countries, fewer than 15% of hospitals have the necessary resources to diagnose and manage DKA adequately, further exacerbating healthcare inequalities (^3^).

In addition to the economic burden, DKA carries severe prognostic implications. A previous episode of DKA significantly increases the risk of all-cause mortality, major adverse cardiovascular events, and advanced microvascular complications, independent of age and sex (^4^). These findings underscore the importance of preventive strategies, particularly for vulnerable populations.

Social determinants of health shape diabetes outcomes, affecting access to healthcare, disease management, and complication rates. Socioeconomic status, healthcare access, and education are closely associated with diabetes-related health disparities (^5^). In Brazil, healthcare services are divided between the public and private sectors. The public system provides free and universal healthcare to all citizens, funded primarily by taxes. It offers broad coverage in principle, yet in practice, resource limitations and high patient demand often undermine access to specialized care and diabetes management. In contrast, the private sector provides faster access to specialists and advanced technologies. However, the higher cost of these services limits their accessibility to those without private health insurance or the ability to pay out-of-pocket (^6^).

Risk factors for DKA include both modifiable and non-modifiable conditions. Non-modifiable factors frequently associated with recurrent DKA include young age, female sex, social disadvantages, and migration history. Conversely, elevated glycated hemoglobin (HbA1c) levels, poor treatment adherence, mental health issues, substance abuse, and low-quality diabetes care are modifiable factors that can be addressed through targeted preventive interventions (^7^).

This study aims to evaluate the factors associated with knowledge of diabetic ketoacidosis among individuals with type 1 diabetes mellitus and to analyze how sociodemographic, clinical, and healthcare access variables influence understanding of the condition’s prevention and management. By addressing knowledge gaps and preventive practices, this study seeks to provide insights for developing strategies to prevent DKA and mitigate its impact on already strained healthcare systems.

## METHODS

This cross-sectional observational study utilized primary data collected via an online questionnaire to evaluate knowledge of DKA among individuals with T1DM receiving care in public and private hospitals in Brazil.

Participants were recruited through Instagram and online groups dedicated to individuals with T1DM. Since the invitations were posted in open groups, the total number of individuals who received the questionnaire link could not be determined. Of the 490 individuals who accessed the questionnaire, 18 declined to participate, and 7 provided incomplete responses, resulting in a final sample of 465 participants.

The structured questionnaire hosted on Google Forms covered topics such as disease history, type of treatment, knowledge of DKA (signs, symptoms, and preventive practices), use of ketone meters, and socioeconomic and educational characteristics.

Data were analyzed using the Jamovi software (version 2.3, Jamovi Project, Australia) (^8,9^). Variable normality was tested with the Shapiro-Wilk test. Differences in scores across income level, treatment modality, and hospital type (public or private) were examined with the Kruskal-Wallis test. Pairwise differences underwent additional analysis using the Dwass-Steel-Critchlow-Fligner multiple comparison test, with p adjusted for multiple comparisons. A significance threshold of p < 0.05 was adopted. Confidence intervals and effect sizes were calculated to quantify the results. All participants provided informed consent before completing the online questionnaire.

## RESULTS

**Figure 1:**
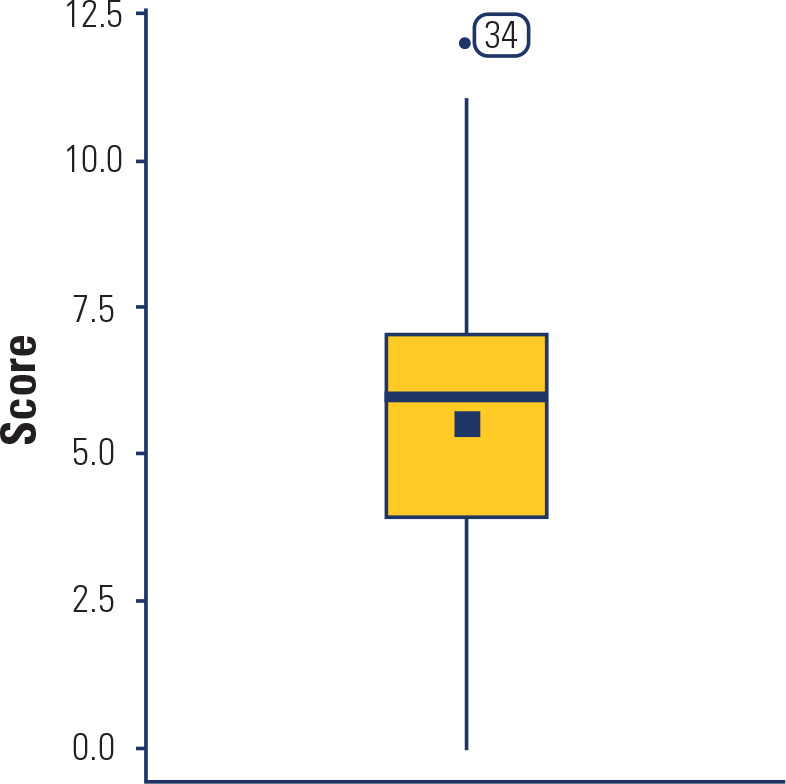
Box-plot score distribution.

The mean age of participants was 33 years (standard deviation [SD] = 10.19), and the mean duration of diagnosed T1DM was 16.41 years (SD = 11.17). Detailed sociodemographic and clinical characteris-tics of the participants are presented in **table 1**. The results showed considerable variability, with a median score of 6.0 out of 13.0 (SD = 2.45; **Figure 1**). Item level accuracy rates ranged from 18.5% to 73.3% (**Table 2**).

**Table 1:** Sociodemographic and clinical characteristics of the participants

Variables	%
Female sex	80
Level of education	
Up to high school	20.6
Undergraduate degree	75
Formal training in the health field	25.6
Monthly income	
3-6MWs^*^	31
1-3MWs	28.4
> 10 MWs	12.3
History of DKA episode	
At least one	54.6
Unaware of previous episode	15.3
Possess a ketone meter at home	22.2
Last HbA1c ≥ 8% or did not know	35
Uses insulin pump as primary treatment	27.9
Received medical guidance during consultations	52
Uses private healthcare services	52.7

* The Brazilian MW stands at R$ 1,518.00 per month, which is approximately US$ 303.60.

MW: minimum wage; DKA: diabetic ketoacidosis; HbA1c: glycated hemoglobin.

**Table 2:** Participants’ accuracy rate in the questionnaire on knowledge of diabetic ketoacidosis

Item	Correct answer	%
What value is considered normal ketonemia (ketones in the blood)?	0.6 mmol/L	18.5
Which of the following situations is called a “sick day” for someone with type 1 diabetes?	Vomiting or nausea and inability to eat	42.2
Ketones are…	All of the above	49.9
What is true about ketones?	They occur when insulin is insufficient in the body	58.5
Why is DKA classified a medical emergency?	Ketones acidify the blood and impair organ function	55.7
What are the symptoms of DKA?	Vomiting, stomach pain, and difficulty breathing	26.0
When ill, how often should someone with diabetes test blood sugar levels?	Every 2-3 hours until blood sugar values fall below 300	28.2
What is NOT good to eat or drink when feeling nauseous and blood sugar levels are low?	Chocolate milk	48.4
Under what circumstances should insulin administration be discontinued?	Never	58.1
All of the following are indications for ketone testing EXCEPT:	A single blood sugar reading > 300 mg/dL	23.2
When using an insulin pump, what should someone with diabetes do FIRST when ketones and blood sugar are above 300 mg/dL in the morning?	Administer a correction dose with a pen or syringe and replace the pump cannula site	30.1
What is the initial intervention when morning blood sugar is 300 mg/dL, ketone levels are low, and nausea is present?	Administer the morning insulin and reassess glucose and ketones after 2 hours	73.3
A person with diabetes should call or go to the emergency room if…	All of the above	66.5

DKA: diabetic ketoacidosis.

Effect-size estimates identified variables associated with better outcomes (**Table 3**). Multiple comparisons were carried out to clarify these associations, highlighting key findings and the impact of the analyzed variables (**Table 4**). Family income influenced performance only when comparing participants earning 6 to 9 minimum wages (14%) with those earning ≤ 1 minimum wage (11.4%; p = 0.02; as of 2025, the Brazilian minimum wage stands at R$ 1,518.00 per month, which is approximately US$ 303.60 at an exchange rate of R$ 5.00 to US$ 1.00). For the remaining data, all significant findings had p < 0.01. Participants treated in the public system (47.3%) showed the worst performance (median score of 5.0), whereas those who used private healthcare (median score of 6.0).

**Table 3:** Kruskal-Wallis test scores by variable

Variable	χ^2^	dl	p-value	ɛ^2^
Monthly income	14.7	5	0.01	0.0318
Healthcare system used	8.03	1	< 0.01	0.0173
Treatment adopted	61.9	3	< 0.01	0.133
Knowledge of DKA	77.1	2	< 0.01	0.166
Guidance received	81.3	4	< 0.01	0.175
Method of measuring ketones at home	36.2	2	< 0.01	0.0779
HbA1c levels during testing	30.5	4	< 0.01	0.0656

ɛ^2^ indicates the effect size, which measures the variance proportion in scores. Values closer to 1 indicate a stronger effect.

dl: degrees of freedom; DKA: diabetic ketoacidosis; HbA1c: glycated hemoglobin.

**Table 4:** Multiple comparisons Dwass-Steel-Critchlow-Fligner comparison scores by variable

Variable	Comparison	M1	M2	W	p-value	MD (%)
Monthly income	6-9 MW versus up to 1 MW	6.0	5.0	4.381	0.02	+ 32.5
Healthcare system used	Private versus public	6.0	5.0	-4.01	< 0.01	+ 12.6
Treatment adopted	Pancreas pump versus scheme	7.0	4.0	-9.73	< 0.	+ 62.9
Knowledge of DKA	Knows versus does not know	6.0	2.0	7.71	< 0.01	+ 206.7
Medical guidance received	During consultations versus never sought	6.0	3.0	12.28	< 0.01	+ 106.3
Method of measuring ketones at home	Blood meter versus none	7.0	5.0	8.49	< 0.01	+ 33.0
HbA1c levels during testing	4-6% versus does not know	6.0	4.0	-4.84	< 0.01	+ 94.1

M: median; MD: mean difference; MW: minimum wage; DKA: diabetic ketoacidosis; HbA1c: glycated hemoglobin.

The type of treatment also showed significant relevance. Participants using artificial pancreas insulin pumps (13.3%) recorded the highest median scores (7.0). Those on slowand rapid-acting insulin with carbohydrate counting (45.6%) had a median score of 6.0 than those on slowand rapid-acting insulin as per prescribed regimen (26.5%; median score of 4.0). Additionally, participants using conventional insulin pumps (14.6%) scored a median of 6.0, showing significance only when compared to those on slowand rapid-acting insulin as per the prescribed regimen. Self reported familiarity with DKA obtained higher scores: participants who knew the condition (86.5%) had a median score of 6.0; those unfamiliar (3.2%) had a median score of 2.0, and those who had heard of it but did not understand its meaning (10.3%) scored a median of 4.0.

Regarding health education on DKA, participants who received medical guidance during consultations (52%) achieved a median score of 6.0, outperforming those who never received guidance (13.1%; median score of 3.0). Furthermore, no significance was observed when compared to those who received advice from friends and family (3.4%), sought information on social media (11.2%), or researched independently through online searches (20.2%).

Participants who had a blood ketone meter at home (22.2%) scored a median of 7.0, exceeding those without adequate means to measure it (77%; median score of 5.0). Urine ketone measurement using test strips (0.9%) exhibited no association. Moreover, glycemic control correlated with knowledge. Participants with HbA1c of 4 to 6% (10.5%) had a median score of 6.0, outperforming those with HbA1c of 8 to 11% (24.9%; median of 5.0), as well as those who were unaware or could not report their last test result (2.8%; median score of 4.0). No significance emerged for HbA1c > 11% (7.3%) or 6 to 8% (54.4%).

Factors such as family income, type of treatment used, and prior knowledge of DKA were associated with higher scores on the questionnaire. The findings demonstrated that patients who received medical guidance during consultations achieved higher scores, as did those who reported HbA1c levels of 4 to 6% and those who stated they were already familiar with DKA.

## DISCUSSION

Socioeconomic status is a key determinant of health, and economically disadvantaged individuals are understood to face a greater risk of DKA episodes (^9-12^). On a broader scale, in countries with lower Human Development Index, economic conditions are also reflected in higher DKA rates (^1^). This study identified differences in the performance on the questionnaire between individuals earning ≤ 1 minimum wage and those earning three to nine minimum wages. However, this factor may require further exploration, as the effect size was small, and no association could be established among participants with higher incomes or those reporting no income.

The healthcare system also influences diabetes health education. In the United States, lack of health insurance or reliance on Medicaid are strong predictors of DKA readmissions (^10,14,15^). Conversely, countries with public healthcare systems divert resources toward acute care over preventive care (^16^). In this study, individuals using the private healthcare sector demonstrated a greater understanding of their condition, potentially due to the quality of services offered. Additionally, individuals who use public healthcare often belong to lower-income groups, which may have influenced their performance on the questionnaire. This potential bias reinforces the need to enhance health education in the public sector, focusing on prevention and strengthening the relationship between patients and healthcare teams. Nonetheless, the small effect size of the healthcare system on performance identified an opportunity to promote equitable care.

Diabetes management tools such as blood glucose monitoring, insulin pump use, and intensive education on proper management reduce the risk of DKA (^7,17^). Notably, individuals using insulin pumps, possessing ketone meters at home, or achieving better HbA1c control performed better on the questionnaire. The need for more rigorous training with these devices likely enhances knowledge and disease management. Ketone monitors are a robust approach to preventing DKA crises (^18^). However, accuracy was poorest on items addressing when to measure ketones and the ketonemia threshold, suggesting lower adherence to device use and inadequate professional guidance.

Recognizing DKA symptoms and proper diabetes management lowers episode frequency (^7,13^), even during the first occurrence, when the individual is unaware of their T1DM diagnosis (^15,19^). In this study, specific DKA symptoms were frequently misinterpreted, with low accuracy rates, highlighting the need to improve access to information and disease monitoring. Prior research shows that children with a DKA event were less likely to have visited an endocrinologist within the preceding 120 days (^20^). Similarly, T1DM patients who received medical guidance during consultations performed better on the questionnaire, supporting evidence that less frequent attendance at medical appointments is associated with recurrent DKA episodes (^7,21^). Finally, as expected, individuals with prior knowledge of DKA achieved higher scores, aligning with the study’s hypotheses.

In conclusion, this study demonstrated that access to insulin pump use, possession of ketone meters, medical guidance, and glycemic control directly influence knowledge of DKA. Although family income and healthcare system utilization showed minimal effect sizes, this pattern aligns with previous research. These findings underscore the potential of targeted educational interventions, expanded access to technology, and stronger relationships between patients and healthcare workers to enhance care and prevent DKA episodes.

This study has several strengths. First, it provides a valuable assessment of knowledge of DKA among individuals with T1DM and identifies key factors associated with better understanding and disease management. Additionally, analyzing healthcare system utilization, socioeconomic status, and disease management practices offers a comprehensive perspective on the determinants of DKA knowledge. Nevertheless, the study sample does not fully represent the Brazilian population with T1DM, as many respondents relied on private healthcare systems or insulin pumps, potentially influencing their knowledge. Additionally, self-reported data may introduce recall bias or social desirability bias, and the cross-sectional design prevents causal inference. Future research should aim for a more diverse and representative sample and incorporate longitudinal approaches to evaluate the impact of educational interventions on DKA prevention.

## Data Availability

the datasets used and/or analyzed in this study are available from the corresponding author upon reasonable request.

## SUPPLEMENTARY MATERIAL

### Control and Knowledge of Ketoacidosis in Individuals Over 18 Diagnosed with Type 1 Diabetes and Caregivers

This form aims to investigate knowledge about ketoacidosis, disease history, and control methods used.

**E-mail**:

**INFORMED CONSENT FORM (ICF**)

You are invited to participate voluntarily in a scientific research study. If you do not wish to participate, there is no problem at all. You do not need to explain why, and there will be no penalty for your decision. You have the full right to decline participation in the study; simply select the corresponding option at the end of this page. You may also refuse to answer any question by stopping the questionnaire, and your responses will not be saved.

To confirm your participation, you must read this entire document and then select the corresponding option at the end. This document is called the ICF (Informed Consent Form). It contains the main information about the study, objectives, methodologies, risks, benefits, and other relevant details.

This ICF refers to the research project “Knowledge of Ketoacidosis in Brazil and Education on Sick Days in Patients with Type 1 Diabetes,” which aims to investigate self-knowledge about diabetic ketoacidosis and diabetes, the use of ketone monitors, and rates related to the prevention of diabetic ketoacidosis.

To obtain a copy of this ICF, you must print it or generate a PDF copy to keep on your computer. You may also request a version of this document at any time via email from the researchers listed at the end of this form.

The research will be conducted through an online questionnaire consisting of approximately 30 questions. It is estimated that you will need about 30 minutes to complete it. The accuracy of your responses is crucial to the quality of the research. The questionnaire will be available for responses from January 1 to April 30, 2023. You will not be compensated, as participation in this research is entirely voluntary.

If you decide to withdraw from the study, you may interrupt the questionnaire and leave the research at any time, without any restrictions or penalties. The risks of the study are not predictable as it consists solely of answering a questionnaire. The benefit of the research is to contribute to the reduction of hospitalizations by understanding the indicators that lead to such situations.

The researchers guarantee and commit to maintaining the confidentiality of all information provided by you in this study. Additionally, data processing will comply with the General Data Protection Law (LGPD - Law 13.709/18). You are entitled to reimbursement for any expenses proven to be related to your participation in the study, as well as to compensation for any damages, in accordance with the law.

This research has been approved by the Research Ethics Committee (REC) of Universidade Iguaçu. If you have any questions about the study, or for complaints and/or suggestions, the Ethics Committee is available at: Av. Abílio Augusto Távora, No. 2134, Block A - 1st floor - Room 103, Nova Iguaçu, RJ. Service hours: Monday to Friday, from 9:00 AM to 12:00 PM and from 1:00 PM to 4:00 PM. Phone: (^21^) 2765-4000. You may also contact via email: cepunigcampus1@gmail.com or cep@campus1.unig.br

To contact one of the study researchers, you may send an email, call, or message them via WhatsApp at any time:

**Principal Investigator:** Rodrigo Siqueira, (Tel. 21 99758-1150), email: rodrigoendocrinologista@gmail.com

**Assistant Researcher(s):** Renan Bruno Faria Pisani, (Tel. 21 99943-70779), email: renanbfpisani@gmail.com

CONSENT TO PARTICIPATE

Phone Number:

**I agree to voluntarily participate in this study as a participant. The researcher has informed me about everything that will happen in the study, what I will need to do, including the possible risks and benefits involved in my participation. The researcher assured me that I could withdraw from the study at any time without explanation and that this decision would not result in any penalties or interruptions to my treatment. I was also informed that I should print or generate a PDF of the ICF to keep a copy and that I can request a version via email from the researchers.**

**I declare that I have read the document above (ANNEX 1) and agree to participate in the study.**

I do not agree to participate (stop answering the questionnaire here)

**Are you a person with type 1 diabetes or a caregiver of someone with this condition?**

* I have type 1 diabetes.

I am a caregiver of someone with type 1 diabetes.

ONLY COMPLETE THIS QUESTIONNAIRE IF YOU ARE OVER 18 YEARS OLD AND HAVE TYPE 1 DIABETES.

**1a.0 - Socioeconomic Questions**

**1a.1 - What is your full name?**

**1a.2 - How old are you?**

**a.3 - What is your gender?**

Male

Female

Prefer not to say

**1a.4 - How many people live with you (including children, siblings, relatives, and friends)? * Moro sozinho(a)**

I live alone.

One person

Two people

Three people

Four people

Five or more people

**1a.5 - What is your household monthly income?**

No income

Up to 1 minimum wage (up to R$ 1,212.00)

1 to 3 minimum wages (R$ 1,212.01 to R$ 3,636.00)

3 to 6 minimum wages (R$ 3,636.01 to R$ 7,272.00)

6 to 9 minimum wages (R$ 7,272.01 to R$ 10,908.00)

10 or more minimum wages (more than R$ 12,120.00)

**1a.6 - What is your level of education?**

No education

Incomplete elementary school

Complete elementary school

Incomplete high school

Complete high school

Incomplete higher education

Complete higher education

Postgraduate / MBA / Master’s / Doctorate

**1a.7 - Do you have a background in healthcare?**

Yes

No

**2a.1 - What year were you diagnosed with Type 1 Diabetes Mellitus?**

**2a.2 - Is your treatment provided by the public or private health network?**

Public health network

Private health network

**2a.3 - What type of treatment do you use to treat diabetes?**

Slow and fast insulin (according to the scheme)

Slow and fast insulin (according to carbohydrate concentration)

Conventional insulin pump

Pump with artificial pancreas (android APS or Medtronic 780G)

**2a.4 - Have you ever heard of ketoacidosis?**

No

Yes

Yes, but I don’t know what it is

**2a.5 - Have you ever had an episode of ketoacidosis?**

Yes, at the time I was diagnosed with diabetes

Yes, after being diagnosed with diabetes

Yes, at the time I was diagnosed and at least once more after the diagnosis

I don’t know what ketoacidosis is

No

**2a.6 - How many episodes of ketoacidosis have you had?**

Never had

Once

Twice

3 times or more

I don’t know

**2a.7 - Have you ever received guidance on ketoacidosis and its complications?**

Yes, there is no doctor’s office

Yes, through friends and family

No, I searched on my own on internet search engines

No, I don’t know on social media

I have never received or looked for information on ketoacidosis

**2a.8 - Have you ever measured your blood ketone levels?**

No

Yes

**2a.9 - Do you CURRENTLY have a way to measure ketone levels at home?**

I don’t have

I have a blood ketone meter

I have urine test strips

**2a.10 - What was your blood sugar level during the ketoacidosis episode?**

Less than 100mg/dl

Between 100 mg/dl and 250 mg/dl

Over 250 mg/dl

I don’t know

I’ve never had an episode of ketoacidosis

**2a.11 - What was your last glycated hemoglobin?**

Between 4% and 6%

Between 6% and 8%

Between 8% and 11%

Over 11%

I don’t know

**3.0 - Ketoacidosis knowledge test**

**2a.3 - Qual tipo de tratamento que você utiliza para tratar o diabetes?**

Insulina lenta e rápida (de acordo com o esquema)

Insulina lenta e rápida (de acordo com contagem de carboidratos)

Bomba de insulina convencional

Bomba com pâncreas artificial (android APS ou Medtronic 780G)

**2a.4 - Já ouviu falar em cetoacidose?**

Não

Sim

Sim, mas não sei o que é

**2a.5 - Você já teve algum episódio de cetoacidose?**

Sim, no momento em que fui diagnosticado com diabetes

Sim, após ser diagnosticado com diabetes

Sim, no momento em que fui diagnosticado e, pelo menos, mais uma vez após o diagnóstico

Não sei o que é cetoacidose

Não

**2a.6 - Quantos episódios de cetoacidose você já teve?**

Nunca tive

1 vez

2 vezes

3 vezes ou mais

Não sei

**2a.7 - Já recebeu orientações sobre cetoacidose e suas complicações?**

Sim, no consultório médico

Sim, através de amigos e familiares

Não, procurei sozinho em buscadores da internet

Não, soube pelas redes sociais

Nunca recebi nem procurei informações sobre cetoacidose

**2a.8 - Você já mediu alguma vez a cetona sanguínea?**

Não

Sim

**2a.9 - Você ATUALMENTE tem meio de medir cetona em casa?**

Não tenho

Tenho medidor de cetona sanguínea

Tenho fitas de medição pela urina

**2a.10 - Quanto estava o nível de açúcar no sangue durante o episódio de cetoacidose?**

Menos de 100mg/dl

Entre 100mg/dl a 250 mg/dl

Acima de 250 mg/dl

Não sei

Nunca tive um episódio de cetoacidose

**2a.11 - Qual sua última hemoglobina glicada?**

Entre 4% e 6%

Entre 6% e 8%

Entre 8% e 11 %

Mais de 11%

Não sei

**3.0 - Teste de conhecimento da cetoacidose**

**Table:**

3.1 - What value is considered normal for ketonemia (ketones in the blood)? 0.3	mmol/l
0.4	mmol/l
0.6	mmol/l
1.5	mmol/l
3.0	mmol/l

I don’t know

**3.2 - Which of the following situations is called a “sick day” for someone with type 1 diabetes?**

Being tired and having a headache

Having a temperature of 37.5°C but able to eat

Having a cold with a little cough and runny nose

*Vomiting or nausea and unable to eat

I don’t know

**3.3 - Ketones are:**

Acids that can be dangerous when they accumulate in the blood

A sign that the body doesn’t have enough insulin

Produced by the body when using fat for energy instead of sugar

*All of the above

I don’t know

**3.4 - What is true about ketones?**

*They occur because there isn’t enough insulin in the body

They don’t occur when blood sugar is low or norma

They are caused by overeating

All of the above

I don’t know

**3.5 - What makes diabetic ketoacidosis (DKA) a medical emergency?**

Ketones in the urine can damage the kidneys

*Ketones make the blood acidic, which disrupts organ function

Blood sugar levels above 300 mg/dL can cause long-term probl ems

It may increase the likelihood of hypoglycemia (very low blood sugar)

I don’t know

**3.6 - What are the specific signs that you are in diabetic ketoacidosis (DKA)?**

Urinating more and feeling more thirsty

Headache, cough, and fever

*Vomiting, stomach pain, and difficulty breathing

All of the above

I don’t know

**3.7 - How often should someone with diabetes test their blood sugar levels when they are sick?**

Before meals and at bedtime

Every thirty minutes for a full day

Every 2-3 hours until feeling better

*Every 2-3 hours until blood

sugar is below 300

I don’t know

**3.8 - What should NOT be eaten or drunk when feeling nauseous and blood sugar levels are low?**

Ice pops

Fruit juice or soda

*Chocolate milk

Lollipops

I don’t know

**3.9 - When should someone with diabetes stop injecting insulin?**

When blood sugar is below 100 mg/dL

*Never

When vomiting and unable to eat

When drinking a lot of water

I don’t know

**3.10 - All of these are times to test for ketones, EXCEPT:**

*If your blood sugar level is above 300 mg/dL just once

If blood sugar stays above 300 mg/dL for several hours

With vomiting and blood sugar above 300 mg/dL

With vomiting, regardless of blood sugar level

I don’t know

**3.11 - When using an insulin pump, what should someone with diabetes do FIRST when they have ketones and blood sugar above 300 mg/dL first thing in the morning?**

Increase basal insulin rates and give correction via insulin pump

*Give correction via injection (pen or syringe) and change the insulin pump cannula site

Drink water and exercise

Go to the emergency room

I don’t know

**3.12 - What should someone with diabetes do first if blood sugar is 300 mg/dL, there is a low level of ketones in the blood, and nausea first thing in the morning?**

*Take morning insulin and check blood sugar and ketones again in 2 hours

Call or go to the emergency room immediately

Drink a lot of water and exercise

I don’t know

**3.13 - A person with diabetes should call or go to the emergency room if:**

Ketones are at moderate or high levels

Vomiting persists for 3 hours or more

Blood sugar is very low or very high for several hours

*All of the above

I don’t know
